# Antimicrobial Resistance of *Escherichia coli* from Broilers, Pigs, and Cattle in the Greater Kumasi Metropolis, Ghana

**DOI:** 10.1155/2021/5158185

**Published:** 2021-06-05

**Authors:** Rita Ohene Larbi, Linda Aurelia Ofori, Augustina Angelina Sylverken, Matilda Ayim-Akonor, Kwasi Obiri-Danso

**Affiliations:** ^1^Department of Theoretical and Applied Biology, Kwame Nkrumah University of Science Technology, Kumasi, Ghana; ^2^Animal Health Division, Council for Scientific and Industrial Research-Animal Research Institute, Accra, Ghana; ^3^Kumasi Centre for Collaborative Research in Tropical Medicine, PMB, UPO, Kumasi 00233, Ghana

## Abstract

Globally, resistance to antimicrobial drugs in food animals is on the rise. *Escherichia coli* of livestock, though commensal in nature, serves as reservoir for antimicrobial resistance genes with the potential of disseminating them. This study sought to examine the antimicrobial resistance profiles of *Escherichia coli* in broilers, pigs, and cattle in the Kumasi Metropolis and undertake molecular characterisation of the resistances. Faecal *E. coli* isolates (*n* = 48) were obtained from 10 broiler farms, (*n* = 43) from 15 pig farms, and (*n* = 42) from cattle from the Kumasi Abattoir using standard bacteriological techniques. The Kirby–Bauer disc diffusion method was employed in testing the sensitivities of 133 *E. coli* isolates to 15 antimicrobials. All 48 isolates from broilers presented no resistance to amoxicillin/clavulanic acid and ceftiofur. A 100% resistance to meropenem was observed in pig and cattle isolates. Multidrug resistance (MDR) across animal groups was 95.8% (*n* = 46), 95.3% (*n* = 41), and 64.3% (*n* = 27) for broilers, pigs, and cattle, respectively. Twenty-eight isolates presenting phenotypic resistance to aminopenicillins and cephalosporins were screened for the presence of extended-spectrum beta-lactamase (ESBL) genes by PCR. One isolate from poultry and another from cattle tested positive for the bla_CTX-M_ ESBL gene. There were no positives for the bla_TEM_ and bla_SHV_ ESBL genes. Commensal *E. coli* of food animal origin represents an important reservoir of antimicrobial resistance that transfers resistance to pathogenic and nonpathogenic microbes affecting humans and animals. There is an urgent need to institute routine surveillance for the establishment of the mechanisms and molecular orientation of resistance in these organisms.

## 1. Introduction

The alarming increase in antimicrobial resistance worldwide has triggered responses from major health regulatory bodies. The World Health Organisation (WHO), Food and Agriculture Organisation (FAO), and the World Organisation for Animal Health (OIE) have in recent times laid downstrategic plans to understand and curb the menace which poses a threat to the health and existing drug reserves for treatment of human and animal infections [1, 2]. A number of factors such as misuse of antimicrobial drugs in human and animal husbandry, sewage and waste water treatment plants, effluents from chemical production factories, and the application of animal manure on farm lands have created conditions for the mingling of human pathogens with environmental bacteria enabling gene mobilization and the spread of antimicrobial resistance [3]. However, antimicrobial resistance may exist naturally in populations that have not previously been exposed to antimicrobials [4].

*Escherichia coli*, though a commensal in the gut of humans and livestock, serves as a reservoir for antimicrobial resistance genes as they are exposed to the pressures applied on the gut flora of the organism throughout its lifetime [5]. As a result, these strains may acquire certain resistant genes and/or undergo mutations that may enhance the microbial homeostasis in the intestinal environment [6]. These beneficial changes to the commensal microorganism might pose a risk of transference of resistant genes to humans through the contamination of meat and other animal products with faecal matter. It is important therefore to monitor the effects of antimicrobial use on the development of resistance in commensals in livestock and selected birds.

Several studies have been conducted in Ghana to assess the extent of antimicrobial resistance [7–9], but only a few have looked at antimicrobial resistance in food animals [10]. This study focused on determining the antimicrobial resistance patterns of commensal *E. coli* in the faeces of broilers, pigs, and cattle to commonly used antibiotics in human and animal husbandry in the Kumasi Metropolis.

## 2. Materials and Methods

### 2.1. Study Area and Population

This cross-sectional study was carried out from November, 2014, to February, 2016, in Greater Kumasi in the Ashanti region of Ghana. Due to rapid urbanisation of the city, most farms within the metropolis have moved to the periurban communities. To obtain a reliable representative of the target population, the stratified-sampling method was used to divide the Kumasi area into two strata; thus, farms within the metropolis and farms within the periurban towns are to ensure the proper representation of farms within the city and surrounding towns. To this effect, a list of broiler and pig producers were obtained and randomly selected within the two strata. A total of 10 broiler farms and 15 pig farms were involved in the study. The Kumasi Abattoir was employed in the collection of cattle faecal samples as it represented a larger pool of cattle.

### 2.2. Sample Collection

Three to four fresh droppings from each pen (broilers and pigs) were collected aseptically as a unit into sterile bags. At the abattoir, fresh droppings of cattle kept in a holding pen before slaughter were collected twice a week for three consecutive weeks. All samples were transported under aseptic conditions in a coolbox with ice to the laboratory within 1-2 hours for further analysis.

### 2.3. Isolation and Identification of *E. coli*

Five (5) grams of each faecal sample were inoculated into 50 ml of buffered peptone water for preenrichment and resuscitation of bacteria and allowed to sit on the bench at room temperature for about an hour. Direct streaking of the inoculum was made on MacConkey agar (Oxoid, UK) and incubated at 37°C for 24 hours. Suspected isolates with pink to red colonies were plated on eosin methylene blue agar (EMBA) (Oxoid, UK) and incubated at 37°C for 24 hours. After incubation, isolates with green metallic sheen were subjected to biochemical testing on API 20E (Biomerieux Inc., France).

All confirmed *E. coli* isolates were put through antimicrobial susceptibility testing by the Kirby–Bauer disc diffusion method with reference to the Clinical Laboratory Standards Institute (CLSI, 2015) standards. *Escherichia coli* (ATCC 25922) was used as quality control. Nine antimicrobials were tested in broilers: ampicillin (10 *μ*g), amoxicillin/clavulanic acid (30 *μ*g), streptomycin (10 *μ*g), tetracycline (30 *μ*g), ciprofloxacin (5 *μ*g), nalidixic acid (30 *μ*g), ceftiofur (30 *μ*g), sulphonamide (300 *μ*g), and trimethoprim (5 *μ*g).

Eleven antimicrobials were used in pigs and cattle: ampicillin (10 *μ*g), tetracycline (10 *μ*g), cotrimoxazole (25 *μ*g), cefuroxime (30 *μ*g), chloramphenicol (10 *μ*g), ceftriaxone (30 *μ*g), cefotaxime (30 *μ*g), ciprofloxacin (5 *μ*g), amikacin (30 *μ*g), meropenem (10 *μ*g), and amoxicillin/clavulanic (30 *μ*g). Antimicrobials tested belonged to these classes: penicillins, quinolones, fluoroquinolones, aminoglycosides, macrolides, cephalosporins, phenicols, folate pathway inhibitors, and tetracyclines.

### 2.4. Molecular Characterisation of Resistant Isolates

To determine the resistant genes encoding the observed phenotypic resistance, polymerase chain reaction (PCR) was carried out on cephalosporin and aminopenicillin resistant isolates to detect the presence of existing extended-spectrum beta-lactamases (ESBLs). The ESBL primers used were for bla_CTX-M_, bla_TEM_, and bla_SHV_. Protocols used were as described by Hasman et al. [11].

### 2.5. Data Analysis

Inhibition zones of antimicrobials were interpreted according to CLSI 2015 standards using the WHONET (2015) software. The software was also used to calculate the percentage of multidrug resistance and to determine antimicrobial resistance profiles of isolates. Microsoft Excel (2013) was used to generate other statistical data.

## 3. Results

A total of 133 *E. coli* isolates were obtained from the three animal groups: 36.1% (*n* = 48) was from broilers, 32.3% (*n* = 43) from pigs, and 31.2% (*n* = 42) from cattle.

### 3.1. Resistance Patterns of Broiler Isolates

All (*n* = 48, 100%) isolates from broilers did not show any resistance to amoxicillin/clavulanic acid and ceftiofur (Table 1). The isolates were highly resistant to sulfonamides and tetracycline (95.7% each), with moderate resistance to nalidixic acid (61.7%) and ciprofloxacin (23.4%).

The dominant profile, a combination of ampicillin-streptomycin-tetracycline-sulfonamide-trimethoprim and ciprofloxacin/nalidixic acid, had a total number of 13 isolates representing 25.5% of broiler isolates tested (Table 2).

### 3.2. Resistance Patterns of Pig and Cattle Isolates

Of all the antimicrobials tested against faecal *E. coli* from pigs (*n* = 43), complete resistance (100%) was recorded against meropenem. Ampicillin resistance was recorded among 95.3% of the isolates. No isolate showed resistance to ceftriaxone and amikacin (Table 3). Two profiles, meropenem-ampicillin-cefuroxime and meropenem-tetracycline-ampicillin-cefuroxime, had the highest percentage of 18.6% of pig isolates tested. A total of 17 profiles were observed after testing the faecal isolates against 11 antimicrobials.

*Escherichia coli* from cattle (*n* = 42) presented no resistance to gentamicin and ciprofloxacin. However, ampicillin resistance was 54.8%, whereas meropenem was 100% resistant with tetracycline recording 11.9% resistance (Table 3). 17 profiles were observed in pig faecal isolates (Table 4), whereas cattle isolates had 24 different resistance profiles (Table 5).

### 3.3. Multidrug Resistance (MDR) in Various Animal Groups

**Figure 1:**
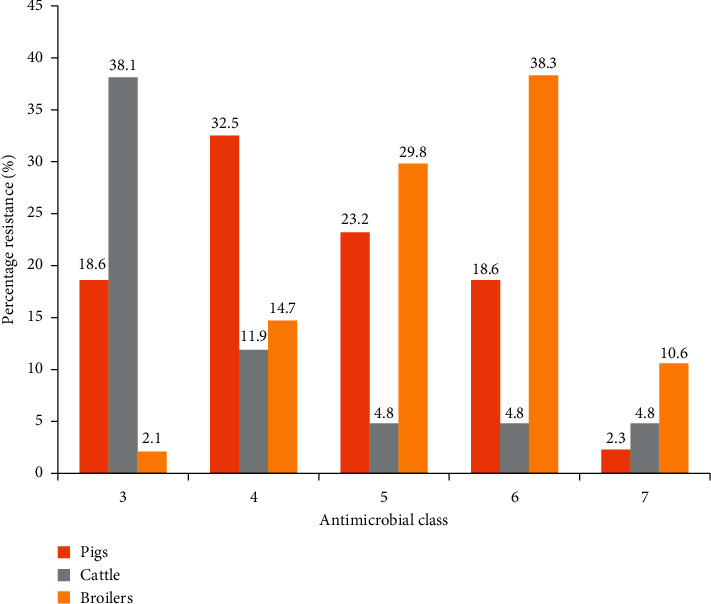
Comparison of percentage multidrug resistance for the three animal groups. Classes tested are penicillins, quinolones, fluoroquinolones, aminoglycosides, macrolides, cephalosporins, phenicols, folate pathway inhibitors, and tetracyclines.

Multidrug resistance (MDR) was defined in our study as resistance to three or more classes of antimicrobials. The three animal groups under study showed distinct patterns of MDR with resistance ranging from three to seven antimicrobial classes (Figure 1). Broilers recorded 95.8% (*n* = 46) MDR, 95.3% (*n* = 41) for pigs, and 64.3% (*n* = 27) for cattle (Figure 1).

## 4. Detection of ESBL Genes in Aminopenicillin and Cephalosporin Resistant *E. coli*

Twenty-eight *E. coli* isolates resistant to ampicillin and cephalosporins were screened for the presence of ESBL genes (CTX-M, TEM, and SHV). The bla_CTX-M_ gene was detected in two (7.1%) isolates, one each from cattle (11.1%) and broiler (10%). There were no positive isolates for the bla_SHV_ and bla_TEM_ genes tested.

## 5. Discussion

Commensal populations of *E. coli* are well known to be enriched reservoirs of genetic material made available to nonpathogenic and pathogenic bacteria to pool from [12], posing a threat by way of the development of antimicrobial resistance in animals and humans [13]. In this study, a number of commonly used antimicrobials in humans and animals recorded high resistances.

The tetracyclines recorded a significant level of resistance across all food animal groups in the study. *Escherichia coli* from broilers showed a resistance of 95.7%, whiles pigs and cattle showed resistance of 44.7% and 11.9% respectively. Globally, resistance in the penicillins, tetracyclines, and sulfonamides which are longstanding antimicrobials in animal production is prevalent [14]. The story is not much different from what pertains in Ghana where [15, 16] have indicated that tetracycline is the most frequently used antimicrobial in poultry. Therefore, the high level of resistance to tetracycline could be a reflection of the use and misuse of this antimicrobial in poultry.

*Escherichia coli* resistance observed to ampicillin was high across all three animal groups with 95.7% of pig isolates being resistant followed by broiler isolates (80.9%) and cattle isolates (54.8%). The high prevalence may have been as a result of the prolonged use of the drug as well as its long existence in animal husbandry use. There is also the factor of most farmers relying on their experiences as well as inputs from other farmers as compared to patronising the services of veterinary officers [15, 17, 18] which they consider much more expensive in administering drugs to their animals especially poultry birds; hence, the apparent resistance is found in these two classes of antimicrobials. The moderate resistance in cattle could be linked to the infrequent use of antimicrobials, which are mainly employed for therapeutic purposes in the species [19].

Generally, fluoroquinolone resistance was highest in isolates from broilers (23.4%) as compared to those from pigs (2.4%) and cattle (0%). Ciprofloxacin is rarely used in pig farming in Ghana [20]; it is, however, used in the poultry industry. Though not a key antimicrobial drug in animal farming, the high incidence in resistance is a cause for worry because studies have shown a close link between the genomes of certain resistant *E. coli* in humans and those of broilers [21]. Ciprofloxacin and nalidixic acid have in other studies proven to be coresistant; hence, the quinolone-nalidixic acid is used as a screening agent for the fluoroquinolone-ciprofloxacin resistance in most studies. A study linked nalidixic acid resistance by disc diffusion in *Salmonella typhi* strains to high MIC values as much as 10 folds to ciprofloxacin in the same strains [22]. Therefore, it is worthy of note that the resistances presented by ciprofloxacin (disc diffusion) in this study could be comparatively higher when tested with the MIC method, with some sensitive/intermediate isolates possibly showing resistance. Quinolone and fluoroquinolone resistance presented could be attributed to the presence of manifold mutations in the quinolone targets of the isolates [23].

The 3rd generation cephalosporin, ceftiofur, designated by FAO as an antimicrobial purposively for veterinary use [24] recorded no resistance in broiler isolates. An accounting factor could be unavailability of this particular drug in the Ghanaian animal farm production. It was, however, an expectation from the start of the study for the possibility of at least observing some level of resistance to cephalosporins. Cefuroxime, a 2nd generation cephalosporin, has been reported to have some level of resistance in Enterobacteriaceae isolated from food animals in Ghana [19]. Cefuroxime as well as cefotaxime (a 3rd generation cephalosporin) resistance was, however, observed in cattle and pigs [25].

The commonest resistance patterns observed were AMP :  STR : TCY : SSS : CIP : TMP : NAL for broilers, MEM : AMP : CXM for pigs, and MEM : AMP for cattle. The antimicrobial classes existing here are the penicillins, streptomycins, fluoroquinolones, quinolones, folate pathway inhibitors, and 2nd generation cephalosporins. Among these are very important human antimicrobial classes (penicillins, fluoroquinolones, and cephalosporins) as such interaction between these microbial populations and human through food/meat consumption and handling of animals (farmers as well as slaughterers) poses a high risk to the human population considering the transfer of resistant genes by mobile genetic elements. Currently, some studies in Ghana have reported microbial contamination of meat products; this could be a source of community-acquired infection if such products are not properly handled and cooked before consumption [26, 27].

Antimicrobial classes were used in comparing multidrug resistance of the food animal groups, and this was defined as isolates having shown resistance to antimicrobials of three or more different antimicrobial classes. Broilers showed the highest percentage of isolates (95.8%) with multidrug resistance followed by pigs (95.3%) and cattle (64.3%). This finding although lower corresponds to that of Donkor et al. [19] where faecal *E. coli* of poultry origin recorded a 100% multidrug resistance. Similar studies carried out in Nigeria [28], Kenya [29], and other parts of the world have also found multidrug resistant bacteria in food animals that can transfer resistance to humans via the foodchain.

Antimicrobial resistance patterns from cattle generally presented the least prevalence (64.3%) as compared to the two other animal groups in this study. Antimicrobials are mainly used for therapeutic purposes in cattle and for prophylactic purposes as well, but this is stopped mostly after the cattle have been weaned. Their extensive mode of rearing in Africa, which most likely eliminates the easy transfer of resistance factors between animals, could also account for the low resistances. It has been proven in some studies that calves have higher resistance to antimicrobials than cattle [30], and this explains the results obtained, as mature, ready to slaughter cattle were sampled in this study. In spite of this, some key resistances were observed which included resistance to ampicillin (54.8%) and cefuroxime (26.2%) with meropenem recording a 100% resistance to all isolates from cattle tested. Carbapenem resistance of Enterobacteriaceae is emerging in humans in Ghana [31, 32], and with the current identification of these trends in animals, much caution must be taken to reduce the transfer of genes at the human animal interface as little is still known about these mechanisms.

ESBL resistance has been recorded in food animals in most parts of Africa, and the most dominant one is the CTX-M gene [33, 34]. In Ghana, ESBL genes have been isolated from meat [27]. The possible exchange of these genes between humans and animals may complicate the treatment of common infections in both animals and humans leading to the use of last resort of antimicrobial agents.

## 6. Conclusion

The study has revealed a high incidence of resistance to commonly used antimicrobials in animal husbandry and human medicine. The resulting resistance profiles showed patterns that reflect the use and misuse of antimicrobials in animal farming with the least used or nonexistent antimicrobials presenting low or no resistance to antimicrobials. The study has established that commensal *E. coli* of food animals is an important reservoir of antimicrobial resistance presenting different facets of resistance due to exposure to antimicrobials as well as acquisition of resistance genes especially through mobile genetic elements. Increased surveillance in food animals must be ensured to create a better picture of the situation and advice policy makers on their decisions.

## Figures and Tables

**Table 1:** Antimicrobial resistance of faecal *E. coli* from broilers (*n* = 48).

Code	Antimicrobial name	%R	%I	%S
TCY	Tetracycline	95.7	2.1	2.1
SSS	Sulfonamides	95.7	0	4.3
TMP	Trimethoprim	93.6	0	6.4
STR	Streptomycin	89.4	10.6	0
AMP	Ampicillin	80.9	4.3	14.9
NAL	Nalidixic acid	61.7	14.9	23.4
CIP	Ciprofloxacin	23.4	17	59.6
AMC	Amoxicillin/clavulanic acid	0	29.8	70.2
TIO	Ceftiofur	0	2.1	97.9

R, resistant; I, intermediate; S, susceptible.

**Table 2:** Resistance profiles of broiler faecal *E. coli*.

Resistance profile	% isolates (*n* = 48)
AMP; STR; TCY; SSS; CIP/NAL; TMP	25.5
AMP; STR; TCY; SSS; TMP; NAL	17.0
AMP; STR; AMC; TCY; SSS; TMP; NAL	10.6
AMP; STR; AMC; TCY; SSS; CIP/NAL; TMP	10.6
AMP; STR; TCY; SSS; TMP	10.6
AMP; STR; AMC; TCY; SSS; TMP	4.3
STR; TCY; SSS; CIP/NAL; TMP	4.3
STR; TCY; SSS; TMP; NAL	4.3
STR; TCY	4.3
AMP; STR; TIO; TCY; SSS; TMP; NAL	2.1
AMP; STR; AMC; SSS; TMP; NAL	2.1
AMP; STR; AMC; TCY; SSS	2.1
STR; TCY; SSS; TMP	2.1

**Table 3:** Antimicrobial resistance of faecal *E. coli* from pigs (*n* = 43) and cattle (*n* = 42).

Code	Antimicrobial name	%R	%I	%S	%R	%I	%S
MEM	Meropenem	100	0	0	100	0	0
AMP	Ampicillin	95.3	2.3	2.3	54.8	4.8	40.5
TCY	Tetracycline	44.2	16.3	39.5	26.2	33.3	40.5
CXM	Cefuroxime	37.2	53.5	9.3	11.9	14.3	73.8
SXT	Trimethoprim/sulfamethoxazole	11.6	2.3	86	9.5	0	90.5
CHL	Chloramphenicol	9.3	14	76.7	7.1	9.5	83.3
GEN	Gentamicin	7	2.3	90.7	4.8	16.7	78.6
CTX	Cefotaxime	7	30.2	62.8	4.8	2.4	92.9
CIP	Ciprofloxacin	2.3	0	97.7	2.4	0	97.6
AMK	Amikacin	0	4.7	95.3	0	7.1	92.9
CRO	Ceftriaxone	0	4.7	95.3	0	0	100

R, resistant; I, intermediate; S, susceptible.

**Table 4:** Resistance profiles of pig faecal *E. coli*.

Resistance profile	% isolates (*n* = 43)
MEM; AMP; CXM	18.6
MEM; TCY; AMP; CXM	18.6
MEM; SXT; TCY; CTX; AMP; CXM	9.3
MEM; TCY; CTX; AMP; CXM; CHL	9.3
MEM; TCY; CTX; AMP; CXM	9.3
MEM; TCY; AMP; CXM; CHL	4.7
MEM; GEN; AMP; CXM	4.7
MEM; AMP	4.7
MEM; CTX; AMP; CHL	2.3
MEM; SXT; AMP; CXM	2.3
MEM; SXT; TCY; AMP	2.3
MEM; AMK; AMP; CXM	2.3
MEM; TCY; GEN; AMP; CXM	2.3
MEM; CIP; AMP; CXM; CHL	2.3
MEM; GEN; CTX; CRO; CXM; CHL	2.3
MEM; TCY; CTX; CRO; AMP; CXM	2.3
MEM; AMK; TCY; CTX; AMP; CXM; CHL	2.3

**Table 5:** Resistance profiles of cattle faecal *E. coli*.

Resistance profile	% isolates (*n* = 42)
MEM; AMP	14.3
MEM	9.5
MEM; CXM	9.5
MEM; AMP; CXM	9.5
MEM; TCY; CXM	7.1
MEM; CXM; CHL	4.8
MEM; TCY; AMP	4.8
MEM; CTX; AMP; CXM	4.8
MEM; TCY	2.4
MEM; AMP; CHL	2.4
MEM; CRO; AMP	2.4
MEM; CTX; CXM	2.4
MEM; TCY; CTX	2.4
MEM; AMK; CXM	2.4
MEM; AMP; CXM; CHL	2.4
MEM; GEN; AMP; CXM	2.4
MEM; TCY; AMP; CXM	2.4
MEM; AMK; AMP; CXM; CHL	2.4
MEM; AMK; CTX; AMP; CXM	2.4
MEM; SXT; GEN; CTX; AMP; CHL	2.4
MEM; SXT; TCY; CTX; AMP; CXM	2.4
MEM; SXT; TCY; CTX; AMP; CXM; CHL	2.4
MEM; SXT; TCY; GEN; CTX; AMP; CXM	2.4

## Data Availability

All data used in the study are available in the manuscript.
